# Mutations in disordered proteins as early indicators of nucleic acid changes triggering speciation

**DOI:** 10.1038/s41598-020-61466-5

**Published:** 2020-03-11

**Authors:** Sergio Forcelloni, Andrea Giansanti

**Affiliations:** 1 Sapienza University of Rome, Department of Physics, P.le A. Moro 5 00185 Roma, Italy; 2 Istituto Nazionale di Fisica Nucleare, INFN, Roma1 section, 00185 Roma, Italy

**Keywords:** Computational biology and bioinformatics, Evolution

## Abstract

In this study, we analyze the role of different structural variants of proteins in the speciation processes. We separate human and mouse proteomes (taken as a reference) into three previously defined variants of disorder: *ordered proteins* (ORDPs), *structured proteins with intrinsically disordered protein regions* (IDPRs), and *intrinsically disordered proteins* (IDPs). Then, using the representation we call here Forsdyke plot, we study the correlation of DNA divergence with the corresponding protein (phenotypic) divergence in the three variants, comparing human and mouse coding sequences with their homologs from 26 eukaryotes. The parameters of the correlation are related to the speciation process. We find that the three variants of disordered proteins are differently related to the speciation process. Specifically, IDPs phenotypically diverge earlier than ORDPs and IDPRs. ORDPs diverge later but are phenotypically more reactive to nucleotide mutations than IDPRs and IDPs. Finally, IDPRs appear to diverge phenotypically later than IDPs, like ORDPs, but they are prone to accept mutations with rates that are similar to those of IDPs. We conclude that IDPs are involved in the early stages of the speciation process, whereas mutations in ORDPs, once speciation is initiated, accelerate phenotypic divergence.

## Introduction

The speciation process is thought, under the chromosomal hypothesis, to be triggered by the accumulation of mutations that make offspring sterile (because meiosis cannot proceed) in the genomes of prospective parents^1^. In this work, we address the question of whether the genes coding for intrinsically disordered proteins selectively act, in the genomes of a group of mammalian species, as triggering genes of speciation. Intrinsically disordered proteins (IDPs) and proteins with long intrinsically disordered regions (IDPRs) are present in all proteomes, including viruses^2^. Interestingly, the content of IDPs/IDPRs tends to increase with the cellular complexity of organisms^3^; approximately $25\mbox{--}30 \%$ of eukaryotic proteins are predicted to be mostly disordered, and more than half of the eukaryotic proteins have long disordered regions^4^.

Comprehensive analyses suggest that proteins with long intrinsically disordered regions sharing loose packing, low degree of tertiary interactions, and weak compactness are prone to have high evolvability (i.e., the ability to adopt new functions within the same fold or changing the fold)^5,6^. This should be related to the unusual properties of IDPs/IDPRs. Firstly, mutations in disordered regions cause smaller stability changes than those in ordered regions^7^. Secondly, the specific order of amino acids in the sequence of disordered proteins is less conserved than in well-structured ones, and this is related to higher rates of accepted point mutations, insertions, and deletions that maintain enough disorder-promoting residues^8^.

It has been shown that speciation genes are fast-evolving and associated with DNA-binding proteins and transcription factors^9^, features that are quite common among genes encoding intrinsically disordered proteins^10,11^, thus suggesting a central role of these proteins in the speciation process.

Although all the above observations point to IDPs as highly evolvable proteins and to their genes as prospective speciation genes, it is not well explored the question of whether proteins with different percentages of disordered residues differently contribute to the speciation process. To address this question, we rely on the recent operational classification of protein disorder by Deiana *et al*.^11^. Thus, we distinguish three broad protein classes, characterized by different structural, functional, and evolutionary properties^11,12^. Namely: (i) ordered proteins (ORDPs); (ii) structured proteins with intrinsically disordered protein regions (IDPRs); (iii) intrinsically disordered proteins (IDPs). The rationale behind this tripartite classification has been extensively discussed previously^11^. In a nutshell, IDPs enrich only a few classes, functions, and processes: *nucleic acid-binding proteins, chromatin-binding proteins, transcription factors*, and *developmental processes*. In contrast, IDPRs are spread over several functional protein classes and GO annotations, partly shared with ORDPs. Moreover, we have observed that ORDPs and IDPs are more subject to natural selection than IDPRs, whereas IDPRs are more subject to mutational bias^12^. The divergence between two species is straightforwardly represented in what we call here the Forsdyke plot^13,14^ of the correlation, in homologous genes, between the accumulation of mutations in the proteins and in the DNA of the two species. Herein, we compared DNA divergence with the corresponding protein divergence between human and mouse genes encoding for ORDPs, IDPRs, and IDPs and their homologs from 26 eukaryotes. Our results provide evidence that after the primary nucleic acid level difference has triggered the speciation process, specific variants of disorder will contribute before others. Specifically, IDPs phenotypically diverge earlier than ORDPs and IDPRs, whereas ORDPs are pivotal in phenotypic divergence. Interestingly, IDPRs appear to diverge phenotypically later than IDPs, like ORDPs, but they have mutational rates that are similar to those of IDPs.

Overall, our work suggests that the functions of ORDPs are maintained and diverge through an evolution that parsimoniously accepts mutations. In contrast, those of IDPs evolve through a more flexible exploration of chances, which drives speciation.

## Results

### ORDPs, IDPRs, and IDPs are characterized by different rates of evolution

**Figure 1:**
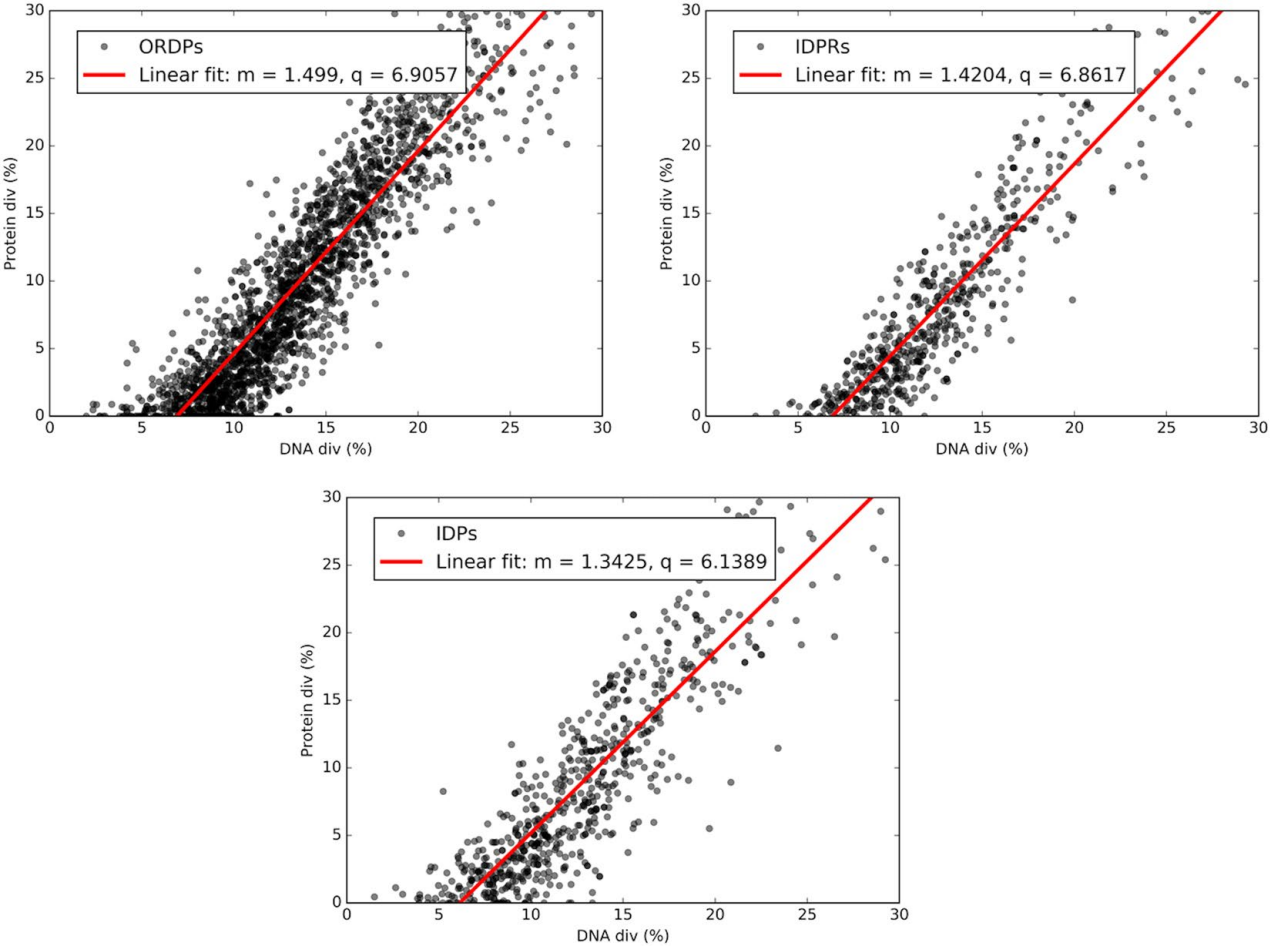
Forsdyke plot. Phenotype (Protein div) vs. nucleotide (DNA div) sequence divergence between human and homologous mouse genes. Each point corresponds to an individual gene. In each panel, we report the best-fit line in red, together with the associated values of the slope (m) and the intercept (q) in the legend.

Human genes were separated accordingly to the variant of disorder of the corresponding proteins (i.e., ORDPs, IDPRs, and IDPs) (see Methods). Each human gene was confronted with the homologous gene in the mouse genome. Each pair of homologous genes is represented by a point in the Forsdyke plot, which correlates protein divergence with DNA divergence. Each point in these plots measures the divergence between pairs of homologous genes in the two species, as projected along with the phenotypic (protein) and nucleotide (DNA) axis.  Fig. 1 is an example of the Forsdyke plot comparing human genes with genes of Mus musculus in the three variants of disorder.

Overall, protein and DNA sequence divergences are linearly correlated, and these correlations can be expressed through slopes and intercepts of the regression lines. To make clear the interpretation of these plots, a few remarks are worth to be placed here. Firstly, the X-axis can be conceived as a time axis because DNA divergence irreversibly increases with time, through the accumulation of both synonymous and non-synonymous mutations^13,14^. Secondly, the X-axis intercept ranges from zero (when divergence was initiated with equal probability at the protein and nucleic acid level) to increasing positive values, reflecting increasing expectations that nucleic acid level differences triggered *initial* divergence. Thirdly, the slope provides an estimate of the fraction of nucleotide mutations that result in protein divergence in the speciation process between two species.

Notably, all the X-axis intercepts in Fig. 1 are positive and significantly different from 0. To statistically validate the differences between the parameters of the linear regressions associated with the variants of disorder, we used a protocol that is described in Methods. All the linear regressions in Fig. 1 are significantly different and, therefore, each variant is characterized by its intercept and slope. Notably, the values of the X-axis intercepts of ORDPs (6.9) and IDPs (6.14) suggest that IDPs require the accumulation of less nucleotide mutations to generate phenotypic divergence, thus triggering speciation earlier than ORDPs; IDPRs, in this respect, are intermediate. In other words, ORDPs are more resistant to nucleic acid changes that will eventually recruit them to play a role in furthering the speciation process.

The steeper slope for ORDPs (1.54) than for IDPs (1.27) indicates that once ORDPs and IDPs are recruited for speciation, then nucleotide changes have a higher probability of inducing amino acid changes in ORDPs than in IDPs. The less steep slope for IDP-encoding genes shows that they are freer to accept synonymous mutations, which are typically neutral and reflect broad mutational processes (extensively reviewed in the introduction of^12^) acting also on non-coding regions.

### Divergence between species is initiated in IDPs and completed in ORDPs

To further confirm these considerations, we extend the study by confronting human coding sequences (separated in ORDPs, IDPRs, and IDPs) with homologs from 26 other mammals. In all the 26 comparisons, the Forsdyke plots are similar to the one shown in Fig. 1, with a well-defined linear relationship associated with each variant of disorder (see Fig. S1). For each comparison, we have checked that the actual slopes ($${m}_{variant})$$ and intercepts ($${q}_{variant})$$ of the linear regressions associated with the three variants of disorder are significantly different according to the test described in the Methods. To visually enhance the differences we subtract, as in a null comparison measurement, averages as follows:

$${\tilde{q}}_{variant}={q}_{variant}-\left(\frac{{q}_{ORDPs}+{q}_{IDPRs}+{q}_{IDPs}}{3}\right)={q}_{variant}-\bar{q}$$

$${\tilde{m}}_{variant}={m}_{variant}-\left(\frac{{m}_{ORDPs}+{m}_{IDPRs}+{m}_{IDPs}}{3}\right)={m}_{variant}-\bar{m}$$

**Figure 2:**
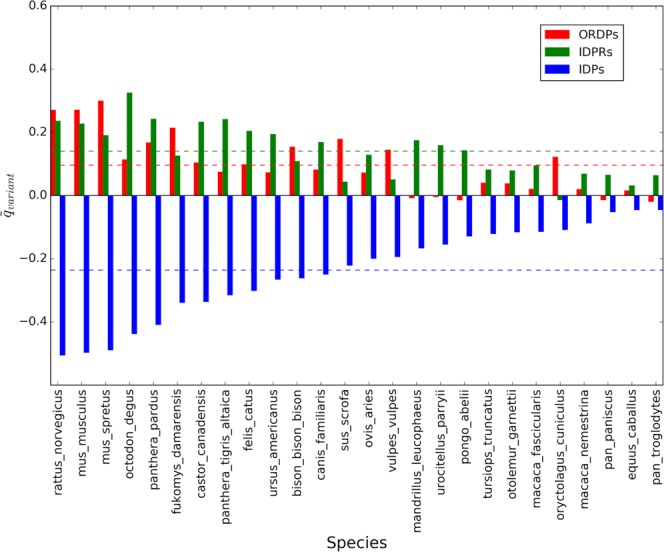
Average corrected intercepts in the Forsdyke plots associated with the variants of disorder, taking Homo sapiens as the reference. On the horizontal axis, we report the 26 eukaryotic species that we considered in the pairwise comparisons. Colored bars are the average corrected intercepts associated with ORDPs (red), IDPRs (green), and IDPs (blue). Horizontal colored lines represent average intercepts of each variant: ${\tilde{q}}_{ORDPs}=\,0.1\pm 0.1,\,{\tilde{q}}_{IDPRs}=\,0.14\pm 0.08$, ${\tilde{q}}_{IDPs}=\,-\,0.24\pm 0.14$.

**Figure 3:**
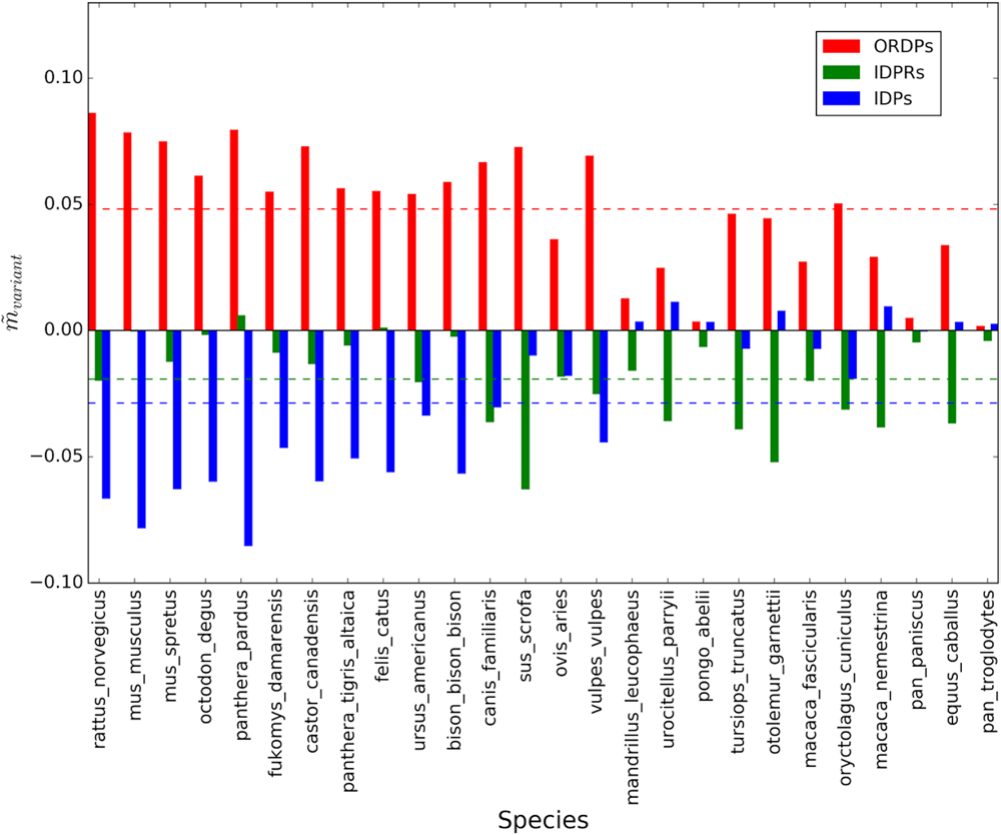
Average corrected slopes in the Forsdyke plots associated with the variants of disorder, taking Homo sapiens as the reference. On the horizontal axis, we report the 26 eukaryotic species that we considered in the pairwise comparisons. Colored bars are the average corrected slopes associated with ORDPs (red), IDPRs (green), and IDPs (blue). Horizontal colored dotted lines represent the average slopes of each variant: ${\tilde{m}}_{ORDPs}=\,0.05\pm 0.02,\,{\tilde{m}}_{IDPRs}=\,-\,0.02\pm 0.02$, ${\tilde{m}}_{IDPs}=\,-\,0.03\pm 0.03$.

Average corrected values of X-axis intercepts $({\tilde{q}}_{variant})\,$and slopes (${\tilde{m}}_{variant})\,$for the 26 comparisons with Homo sapiens are shown in Figs. 2 and 3. Note that the data were ordered following the values of the intercepts.

 In most of the pairwise comparisons shown in Figs. 2 and 3, slopes and intercepts associated with the different variants of protein disorder are evidently different. However, some cases could be controversial (see the species located on the right of the horizontal axis in Fig. 2). This is why we decided to statistically test these differences, in each one of the comparisons, using the protocol indicated in the Methods. Then, we tested the first null hypothesis that the slopes of the regression lines are equal. We found that differences between slopes are very significant ($p-value < 0.0001$) in all the pairwise comparisons, except for *Pan troglodytes*, *Pongo abelii*, *Equus caballus, Urocitellus parryii*, and *Ovis aries* ($\,p \mbox{-} value > 0.05$). In these organisms, having parallel regression lines associated with the three protein variants, we tested for significant differences in the intercepts taking as null hypothesis that the intercepts associated with each variant of disorder are all the same. Following again the significance test on regression parameters we have implemented as in Armitage *et al*.^26^, we found that the intercepts are significantly different ($p \mbox{-} value < 0.05$) in all the 26 pairwise comparisons.

As a general trait, IDPs are characterized, in each one of the comparisons, by the lowest values of the intercepts (as suggested by the blue bars in Fig. 2), thus indicating that IDPs require the accumulation of fewer mutations to be recruited in the speciation process with respect to ORDPs and IDRPs. On the other hand, ORDPs are characterized by the highest values of the slopes (as indicated by the red bars in Fig. 3) in all comparisons, thus suggesting that nucleotide mutations in ORDPs (steeper slopes) play a more relevant role to further progress speciation after recruitment than in IDPs and IDPRs (less steep slopes).

It is worth noting that with closely related (allied) species (i.e., *Pan troglodytes*, *Pongo abelii*, *Mandrillus leucophaeus*, *Macaca nemestrina*, *Macaca fascicularis*, and *Pan paniscus)* the intercept on the X-axis is near zero (see Figs. 2 and S1). This observation suggests that *genic* differences (i.e., amino acid changes) may have played a significant role in the *Pan troglodytes/Pongo abelii/Pan paniscus – Homo sapiens* divergence (i.e., “genic speciation”). In contrast, in the other cases *non-genic* differences (both within and external to genes) may have triggered the separation from the ancestral type into two different species (i.e., “chromosomal speciation”).

### Similar results are found considering Mus musculus as the reference

**Figure 4:**
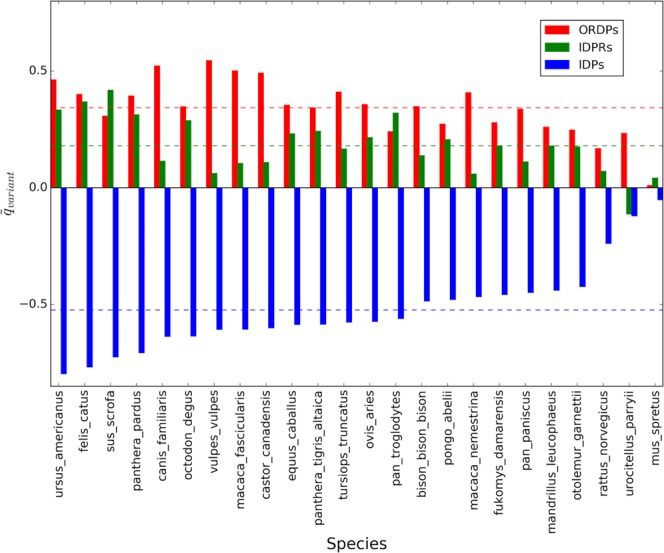
Average corrected intercepts in the Forsdyke plots associated with the variants of disorder, taking Mus musculus as the reference. On the horizontal axis, we report the 25 eukaryotic species that we considered in the pairwise comparisons. Colored bars are the average corrected intercepts associated with ORDPs (red), IDPRs (green), and IDPs (blue). Horizontal colored lines represent the average intercepts of each variant: ${\tilde{q}}_{ORDPs}=\,0.34\pm 0.12,\,{\tilde{q}}_{IDPRs}=\,0.18\pm 0.12$, ${\tilde{q}}_{IDPs}=\,-\,0.52\pm 0.18$.

**Figure 5:**
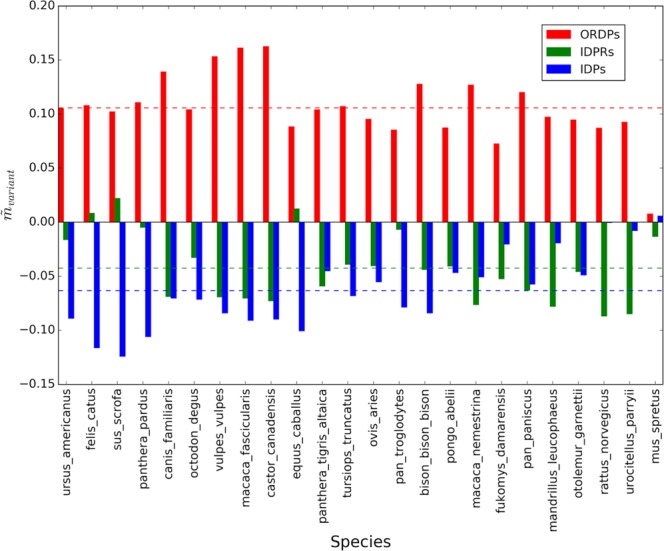
Average corrected slopes in the Forsdyke plots associated with the variants of disorder, taking Mus musculus as the reference. On the horizontal axis, we report the 25 eukaryotic species that we considered in the pairwise comparisons. Colored bars are the average corrected slopes associated with ORDPs (red), IDPRs (green), and IDPs (blue). Horizontal colored lines represent average slopes of each variant: ${\tilde{m}}_{ORDPs}=\,0.11\pm 0.03,\,{\tilde{m}}_{IDPRs}=\,-\,0.04\pm 0.03$, ${\tilde{m}}_{IDPs}=\,-\,0.06\pm 0.03$.

All the results shown above were obtained by taking the genome of Homo sapiens as a reference in pairwise comparisons with other species. To corroborate these observations, we repeated the analyses above with Mus musculus as the reference. Thus, we progressively confronted coding sequences in Mus musculus (separated in ORDPs, IDPRs, and IDPs) with their homologs from 25 eukaryotes. All comparisons produced results similar to Fig. 1 (see Fig. S2). Therefore, for each species put in contrast with Mus musculus and each variant of disorder, we retrieve distinct values of the intercept and slope in the Forsdyke plot. Then, we subtract averages in each comparison for divergence, as we have done in the previous section (Figs. 4 and 5).

Following the procedure described in Methods, we tested the first null hypothesis that the slopes of the regression lines are all equal. We found that differences between slopes are very significant ($p \mbox{-} value < 0.0001$) in all comparisons, except for *Mus spretus* ($p \mbox{-} value > 0.05$). For this organism, we tested the second null hypothesis that the intercepts of the regression lines are the same. We found that the intercepts are significantly different ($p \mbox{-} value < 0.05$).

Similarly to what observed in the previous section, IDPs are characterized by the lowest values of the intercepts (blue bars in Fig. 4), whereas ORDPs are marked by the highest values of the slopes (red bars in Fig. 5). Therefore, we found further evidence that IDPs are more susceptible at the ‘start’ of the speciation process, whereas ORDPs further accelerate the phenotypic divergence once they are recruited.

## Discussion

Intrinsically disordered proteins are expected to play an essential role in fostering protein evolution and in the formation of complex cellular pathways, especially in multicellular organisms.

The focus of this study is on the role of different variants of protein disorder in the speciation process. We have correlated protein divergences and DNA divergences (Forsdyke plots) by comparing coding sequences of Homo sapiens and Mus musculus (associated with ORDPs, IDPRs, and IDPs) and homologous sequences in several other mammals.

On the one side, a positive X-axis intercept in the Forsdyke plots measures both the number of synonymous mutations in the coding regions and the amount of mutational bias in the non-coding regions that should accumulate before non-synonymous protein divergence takes place, initiating speciation. On the other side, the slope of the regression line in the Forsdyke plot is an estimation of the fraction of DNA mutations that result in amino acid substitutions in the speciation process between two species.

It is worth noting that synonymous mutations in coding regions, being non-amino-acid-changing, might be useful indicators of concomitant mutations that occur outside protein-encoding regions^12–14^.

We have shown here that the onset of speciation occurs at different evolutionary times in the genes related to the different variants of disordered proteins. In particular, IDPs require the accumulation of fewer mutations to be recruited in the speciation process with respect to ORDPs and IDRPs. Since the genes related to IDPs are the first to be recruited in the protein divergence process, it is tempting to conceive that these genes could act as sentinel-genes of speciation and that intrinsically disordered proteins are, so to speak, like “*canaries*” in a coal mine, acting as early sensors of genome-wide nucleic acid changes, that eventually ignite speciation. Notably, ORDPs start to differ in amino acids later than the other variants, but then they, having steepest slopes in the Forsdyke plots, are more reactive to nucleic acid changes than IDPRs and IDPs, appearing as the sustainers of the divergence between species, which eventually extends to the proteins that are more structurally constrained.

Noteworthy, both in Homo sapiens and in Mus musculus, IDPRs have slopes more similar to those of IDPs than to those of ORDPs (Figs. 2 and 4); conversely IDPRs have intercepts more similar to those of ORDPs (Figs. 3 and 5). Then, IDPRs appear to diverge later than IDPs, likely due to the structural constraints that they share with ORDPs. Nevertheless, IDPRs accept mutations with rates that are similar to those of IDPs, primarily attributed to the presence of long disordered regions in their structures.

We also note that IDP-encoding genes display less steep slopes in the Forsdyke plot. This is a clear indication that these genes, once the protein divergence is started, are comparatively freer to accept nucleic acid changes, especially synonymous, neutral mutations. This observation confirms the view that intrinsically disordered proteins are the protein sector where evolution is incubated because neutral mutations usually leave the primary function of the molecule unchanged while paving the way for new features to emerge^6,15,16^.

Notably, similar results are found by considering Mus musculus as the reference, thus confirming that the above considerations should have a certain degree of general validity. Also in this case, IDPs appear to be involved in the early stages of the speciation process, whereas ORDPs appear to accelerate the further phenotypic divergence.

Finally, let us speculate that, although the above-commented findings support a “chromosomal” argument suggesting that IDPs might be passive sensors (“canaries”) of genome-wide changes igniting speciation, we cannot exclude an active role of IDPs themselves. The active contribution of IDPs to speciation should be connected to their functional spectrum, enriched by proteins that interact with nucleic acids (e.g., transcription factors)^11^. Now, it is reasonable to admit that proteins interacting with nucleic acids are characterized not only by the basal “chromosomal” mutational rates inherited by the diverging changes in their coding regions, but they are specifically subject to extra mutational rates generated by the necessity (like in a feed-back) to adapt to changing, diverging substrates. Then, we expect that the IDPs specifically involved in the molecular recognition of nucleic acids should display the highest mutational rates among IDPs, contributing to speciation. The higher rate of mutations in some of these genes (e.g., those involved in DNA repair) might lead to directional changes in genomic GC content, fostering differentiation of species^17^. In this line of reasoning, several evolutionary and molecular features have been recently revealed that make transcription factors, especially the KRAB-ZNF family (which are IDPs), reliable candidates to play an important role in speciation^9^.

Overall, our study provides evidence of the different roles played by ORDPs, IDPRs, and IDPs in the speciation process. IDPs appear to be involved in the early stages of the speciation process, whereas ORDPs as pivotal to sustain phenotypic divergence as measured by the rate of amino acid substitutions per nucleotide mutations.

## Methods

### Data sources

The proteomes of Homo Sapiens and Mus Musculus were downloaded from the UniProtKB/SwissProt database (manually annotated and reviewed section - https://www.uniprot.org/uniprot/?query=reviewed:yes)^18^. Coding DNA Sequences (CDSs) of Human and Mus Musculus were retrieved by Ensembl Genome Browser 94 (https://www.ensembl.org/index.html)^19^. Only genes with UniProtKB/SwissProt ID have been included to make sure we only consider coding sequences for proteins. We consider only CDSs that start with the start codon (AUG), end with a stop codon (UAG, UAA, or UGA), and have a multiple length of three. Each CDS was translated into the corresponding amino acid sequence, and then we filter all sequences that do not have complete correspondence with a protein sequence in UniProtKB/SwissProt. Incomplete and duplicated gene sequences with internal gaps, unidentified nucleotides were removed from the analysis. Two lists of 18150 and 14757 CDSs were generated for Human and Mus Musculus, respectively.

### Disorder prediction

We identified disordered residues in the protein sequences using MobiDB3.0 (http://mobidb.bio.unipd.it)^20^, a consensus database that combines experimental data (especially from X-ray crystallography, NMR, and cryo-EM), manually curated data, and disorder predictions based on various methods.

### Classification of the human proteome

The proteomes of Homo Sapiens and Mus Musculus were partitioned into variants of disorder following the operational classification by Deiana *et al*.^11^. We distinguish three variants of human proteins: *i) ordered proteins* (ORDPs): they have less than 30% of disordered residues, no C- or N-terminal segments longer than 30 consecutive disordered residues as well as no segments longer than 40 consecutive disordered residues in positions distinct from the N- and C-terminus; *ii) proteins with intrinsically disordered regions* (IDPRs): they have less than 30% of disordered residues in the polypeptide chain and at least either one C- or N-terminal segment longer than 30 consecutive disordered residues or one segment longer than 40 consecutive disordered residues in positions distinct from the N- and C-terminus; *iii) intrinsically disordered proteins* (IDPs): they have more than 30% of disordered residues in the polypeptide chain.

In a nutshell, ORDPs are then proteins with a limited number of disordered residues and the absence of disordered domains. IDPRs, unlike ORDPs, are proteins with at least one long disordered segment (implying a short half-life^21^) accommodated in globally folded structures. IDPs should include, in this classification, proteins with a significant percentage of disorder, implying an unfolded structure and a high susceptibility to proteolytic degradation^22^.

The rationale behind definitions i) and ii) above, which take into account the sequence location of the long disordered segments, is extensively discussed in^11^.

Also, the rationale behind the choice of the 30% threshold can be extensively discussed (see again^11^). Let us here observe that it has been shown that proteins with more than 30% of disordered residues are more prone to proteolytic degradation^22^, likely due to lack of folding. In line with this argument and in agreement with other works that use the same threshold^23,24^, we have then defined proteins with more than 30% of disordered residues as IDPs.

### Protein and DNA divergence: Forsdyke plot

Inspired by the graphs found in chapter 7 of a book^14^ and in a paper^13^ by D. R. Forsdyke, we have built correlation plots between protein divergences (as measured by amino acid substitutions) and DNA divergences (nucleotide substitutions) in pairwise comparisons of homologous genes in two different, diverging species. These plots were adapted by Forsdyke from a paper by Wolfe and Sharp^25^.

Either the human proteome or the Mus musculus proteome (separated in ORDPs, IDPRs, and IDPs) were taken as a reference to test speciation divergence with 26 other mammalian species. To evaluate divergences, we used the Ensembl genome browser^19^. In particular, for each human (mouse) gene, we used the Ensembl REST API http://rest.ensembl.org (option ‘homology/id/:id’) to retrieve its alignment with a homologous sequence in the genome of the species to be confronted. We compared, in each pair of species, base substitutions in the coding sequences with amino acid substitutions in the corresponding protein sequences. Thus, for each pair of sequences, we calculate the percentage of DNA sequence divergence $(DNA\,div\,( \% ))$ as:

$$DNA\,div\,( \% )=\left(1-\frac{{N}_{match}}{L}\right)\times 100,$$

where ${N}_{match}$ is the number of matches and $L$ is the length of the DNA alignment. The protein sequences were then aligned using the DNA alignments as templates. Then, we evaluate the percentage of protein sequence divergence ($Protein\,div\,( \% )$) as follows:

$$Protein\,div\,( \% )=\left(1-\frac{{N}_{match}}{L}\right)\times 100,$$

where ${N}_{match}$ is the number of matches and $L$ is the length of the protein alignment. Let us point out that here amino acid mutations are considered as a signal of phenotypic divergence leading to speciation. Taking amino acid substitutions as a measure for phenotypic divergence might appear as an oversimplification. Of course, phenotypic divergence is a complex result of evolution, but we believe that the Forsdyke plot offers an effective parametrization useful to elegantly estimate the fraction of genotypic mutations that are likely to produce phenotypic divergence and speciation.

### Statistical tests on the parameters of linear regressions of independent Forsdyke plots

Each one of the 26 species considered in Figs. 2, 3, 4, and 5 were compared either with Homo sapiens or with Mus musculus for evolutionary divergences. The first step in each comparison is to compute the regression line between protein vs. DNA sequence divergence in the Forsdyke plot getting values of intercept and slope for each variant of disorder (ORDPs, IDPRs, and IDPs). To test whether the regression parameters associated with each variant are different or not, we have followed a protocol found in chapter 11 of the classic book by Armitage *et al*.^26^ devoted to the statistical comparison of linear regressions over different datasets. The protocol is based on a double test of hypotheses. The first null hypothesis is that the slopes of the regression lines associated with ORDPs, IDPRs, and IDPs are all equal. If, following an F-statistic test, the ${\rm{p}} \mbox{-} {\rm{value}}\,$is lower than $0.05\,$, we reject the first null hypothesis, and then the regression lines cross each other. At this point, a subsequent statistical test on the difference between X-axis intercepts of two crossing lines consists of checking that the crossing point has an ordinate that is different from 0^27^. If the $p \mbox{-} value$ in the first test is bigger than 0.05, we accept the null hypothesis that the regression lines are parallel, and we then test the second null hypothesis that their intercepts are all the same. If the ${\rm{p}} \mbox{-} {\rm{value}}$ is lower than 0.05, we conclude that the regression lines are parallel but with significantly distinct X-axis intercepts. If the ${\rm{p}} \mbox{-} {\rm{value}} > 0.05$, then there is no evidence that the regression lines are different.

## Supplementary information

Supplementary information.
