# Supplementary material for: Mutations in disordered proteins as early indicators of nucleic acid changes triggering speciation
Source: Sci Rep. 2020 Mar 11;10:4467. doi: 10.1038/s41598-020-61466-5 (PMC7066166; doi:10.1038/s41598-020-61466-5)
Supplement: Supplementary file 1 — Supplementary information. [file 41598_2020_61466_MOESM1_ESM.docx]

**Title page**

## Title: Mutations in disordered proteins as early indicators of nucleic acid changes triggering speciation

**Authors:** Sergio Forcelloni^1*^ and Andrea Giansanti^1,2^

**Affiliation:** ^1^ Sapienza University of Rome, Department of Physics, P.le A. Moro 5, 00185 Roma, Italy. ^2^ Istituto Nazionale di Fisica Nucleare, INFN, Roma1 section. 00185, Roma, Italy.

***Corresponding Author:** Sergio Forcelloni

Sapienza University of Rome, Department of Physics, P.le A. Moro 5, 00185 Roma, Italy.

**E-mail:** [sergio.forcelloni@uniroma1.it](mailto:sergio.forcelloni@uniroma1.it); sergio.forcelloni@gmail.com

**Phone:** +393934073500

**Supplementary information**


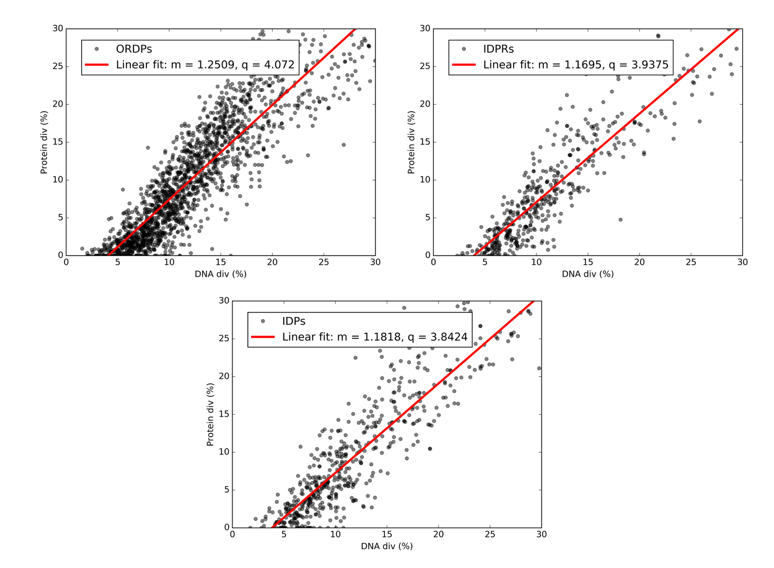

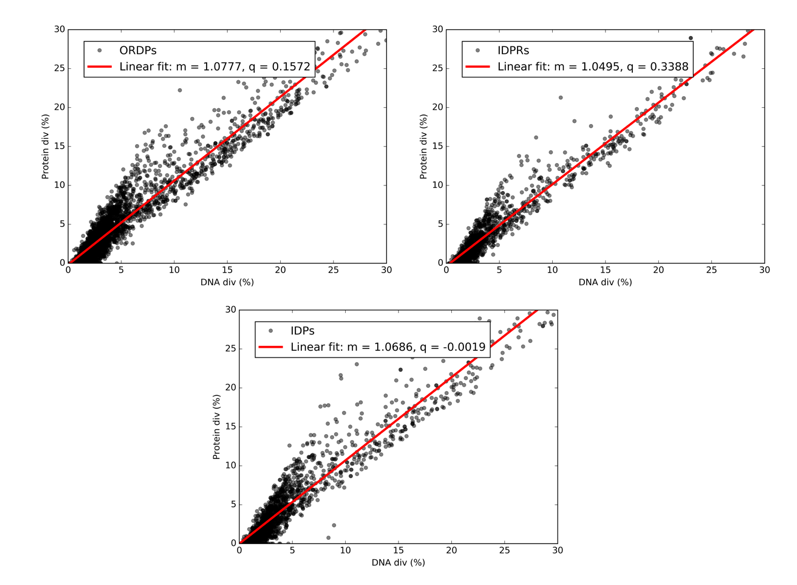


Oryctolagus cuniculus Mandrillus leucophaeus


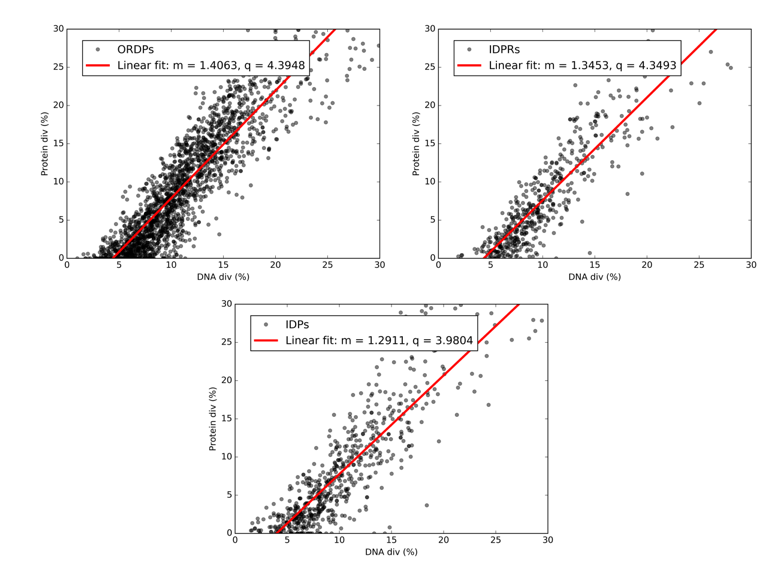

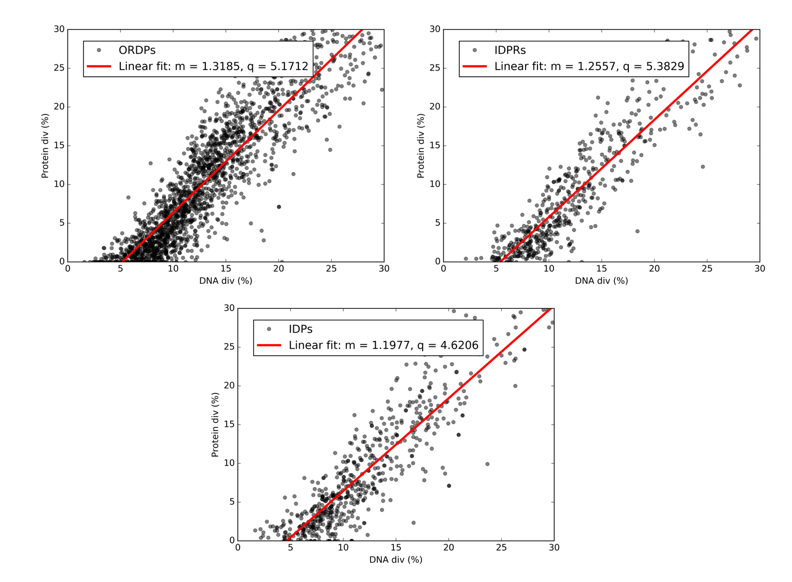
Bison bison bison Octodon degus


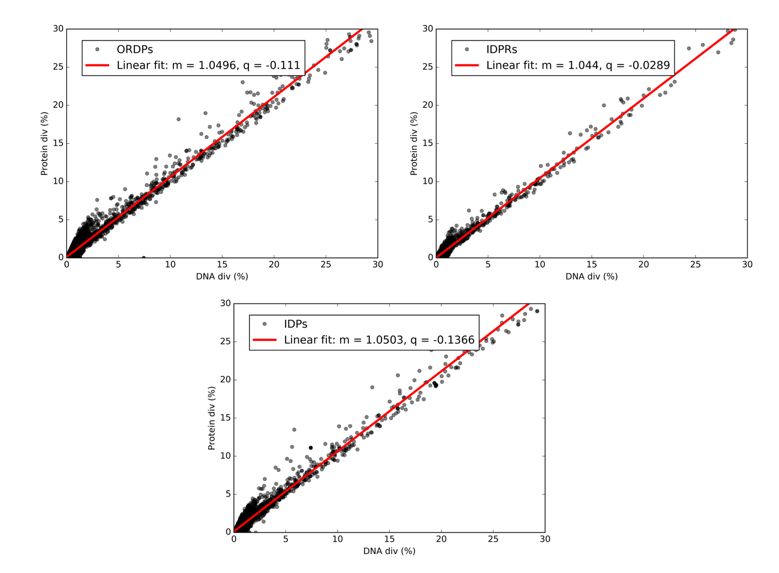

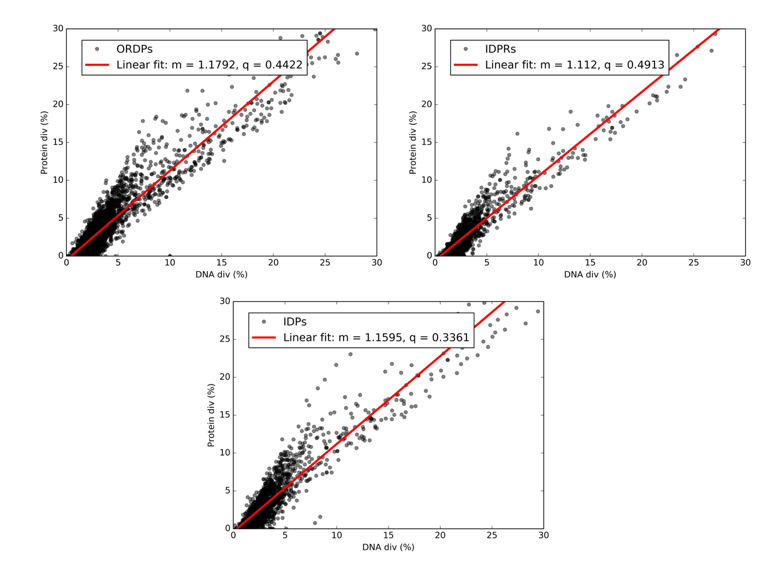


Pan troglodytes Macaca nemestrina


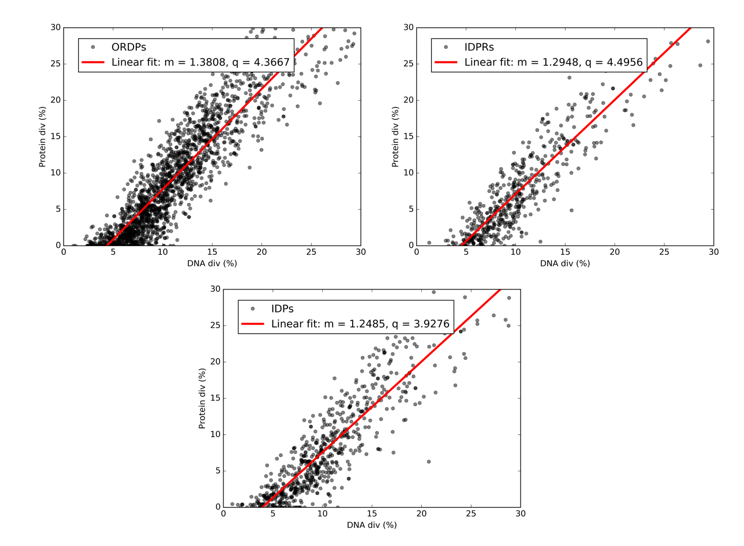

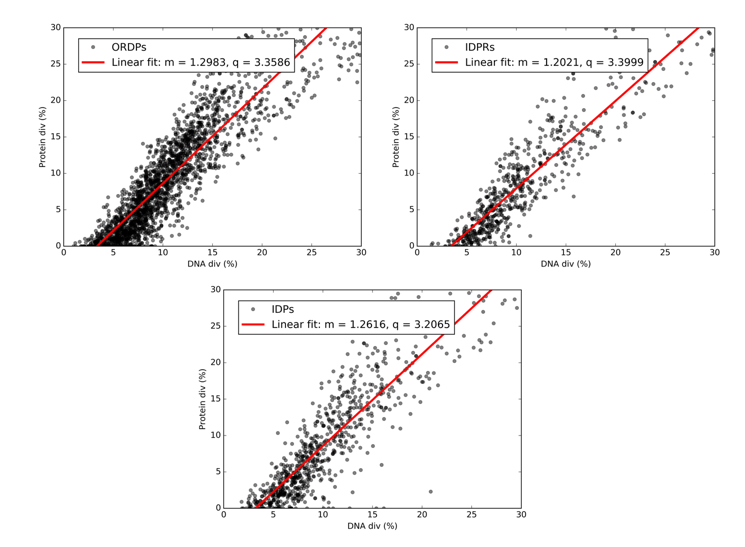


Castor canadensis Otolemur garnettii


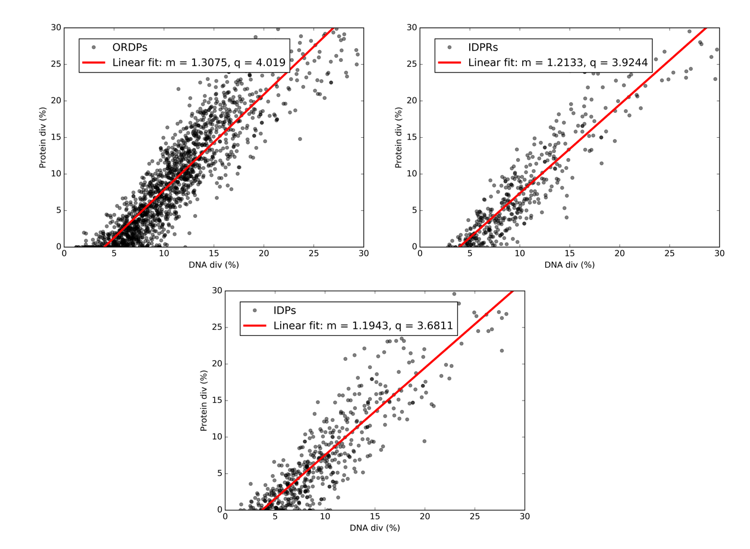

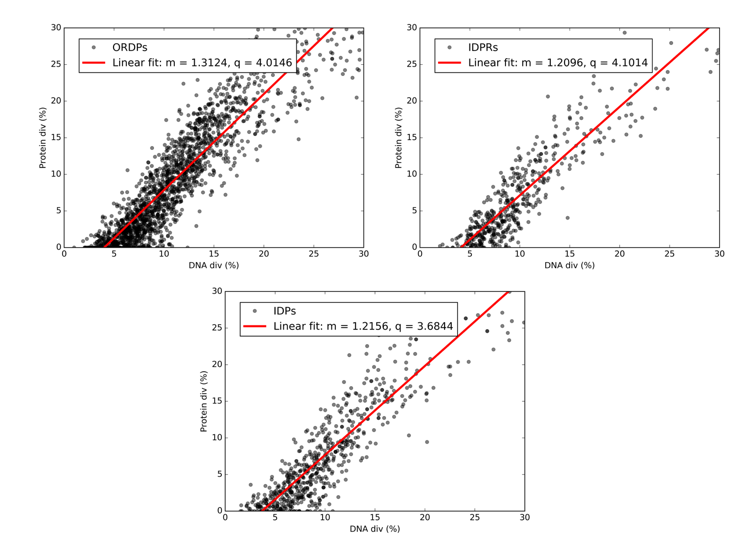


Vulpes vulpes Canis familiaris


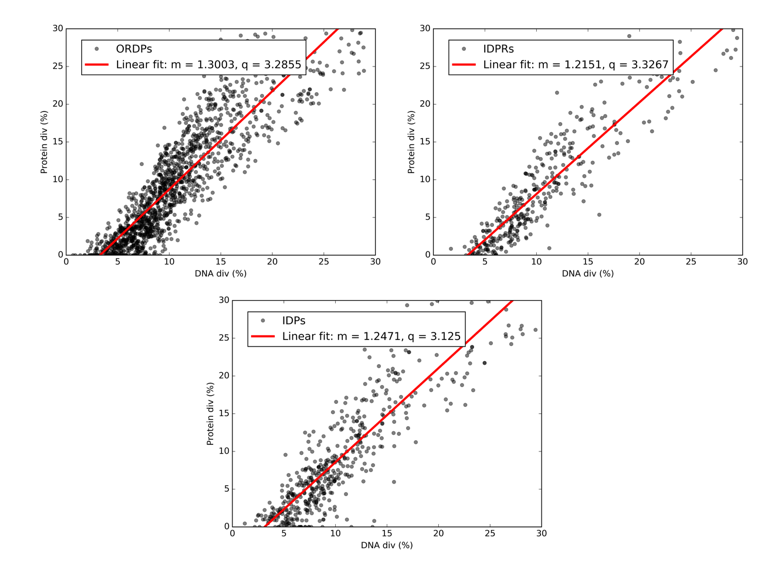

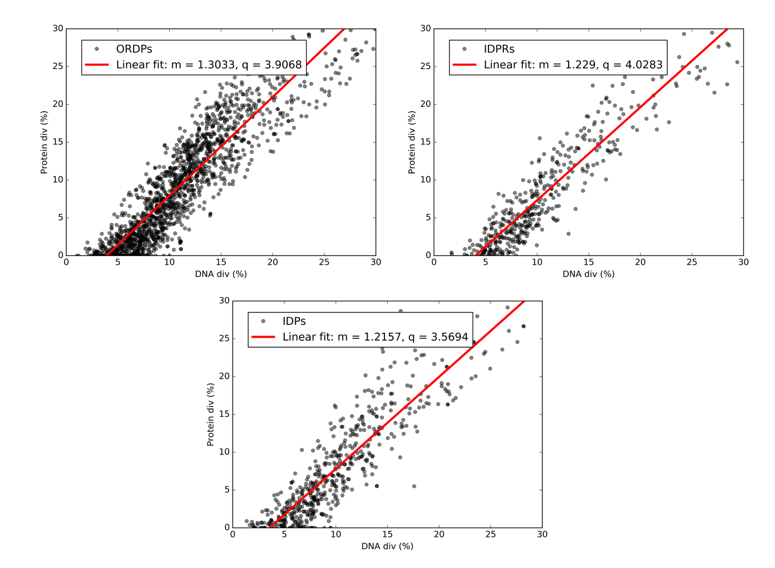


Tursiops truncates Ursus americanus


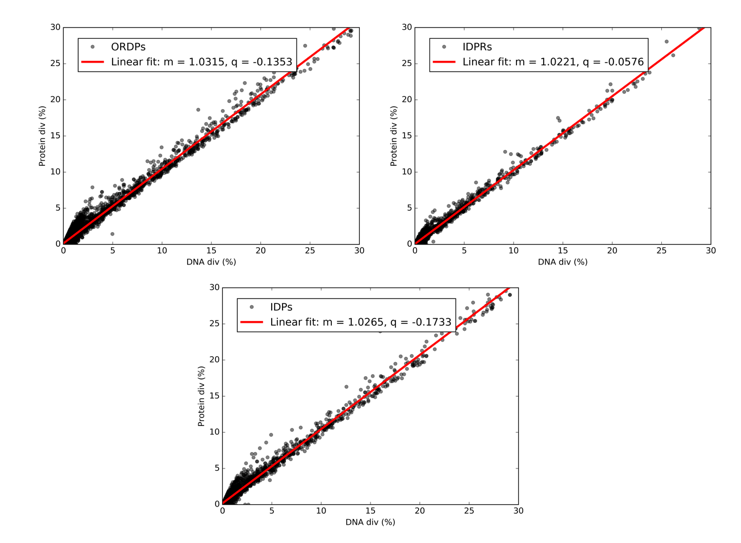

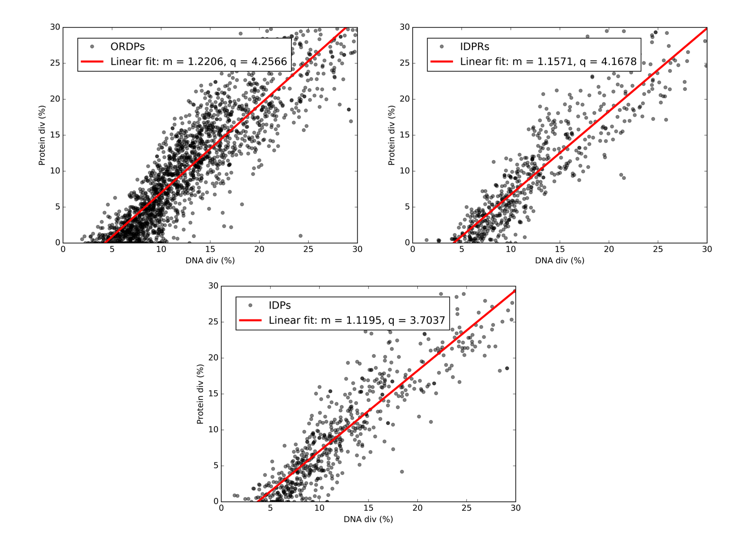


Pan paniscus Fukomys damarensis


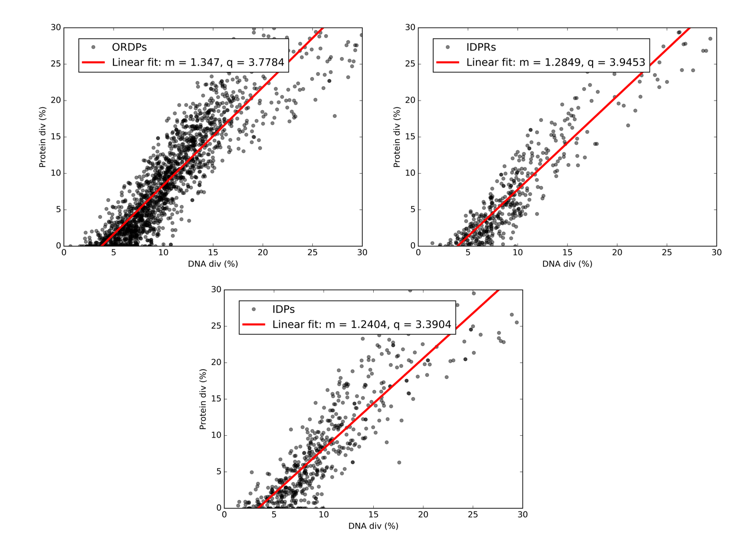

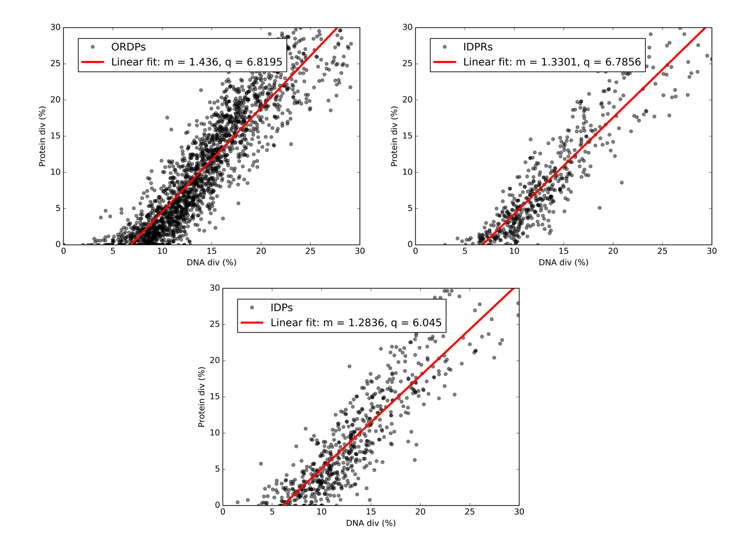


Panthera tigris altaica Rattus norvegicus


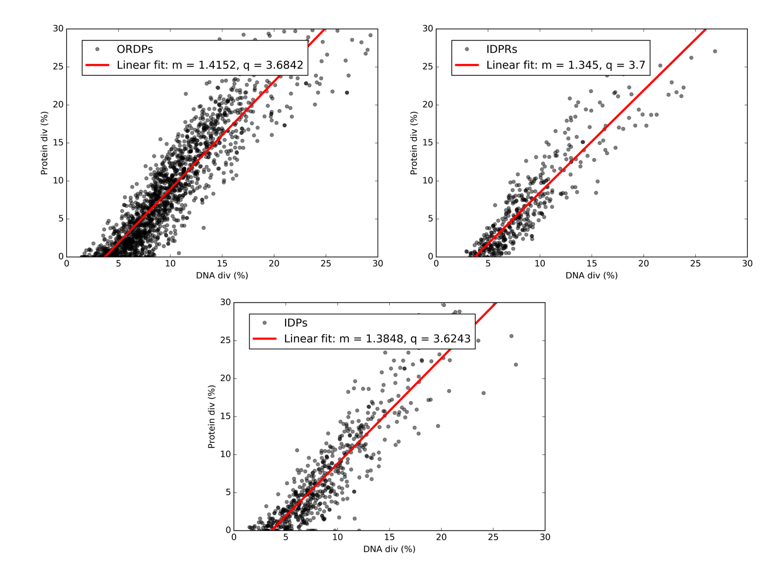

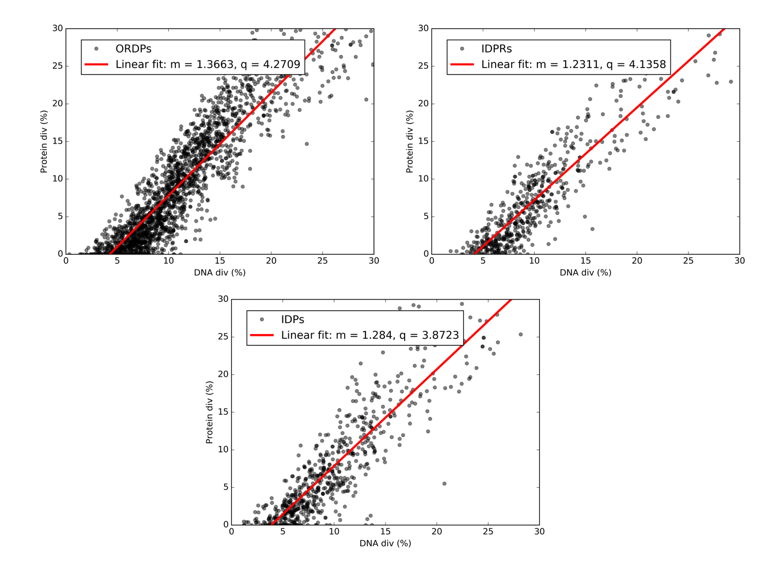


Equus caballus Sus scrofa


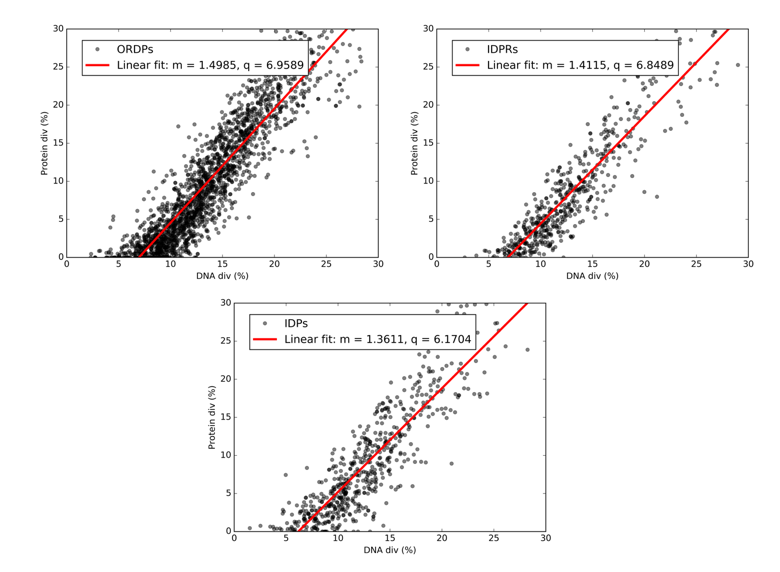

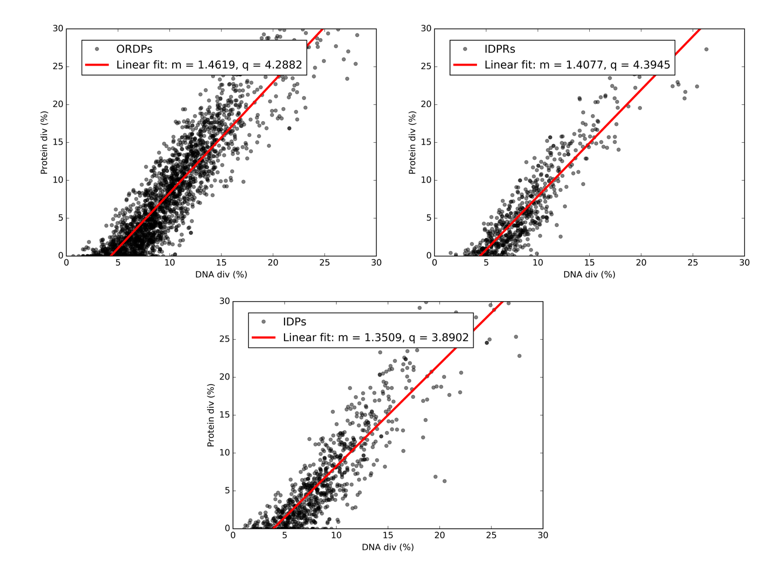


Mus spretus Felis catus


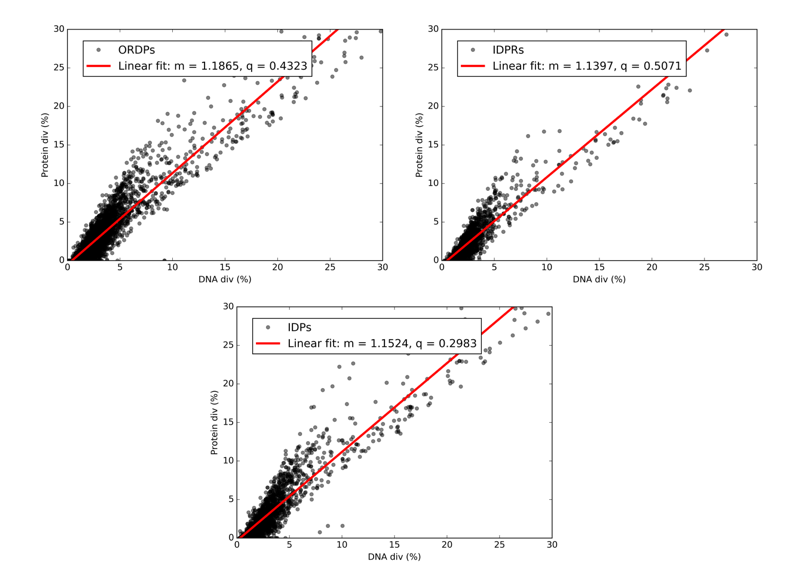

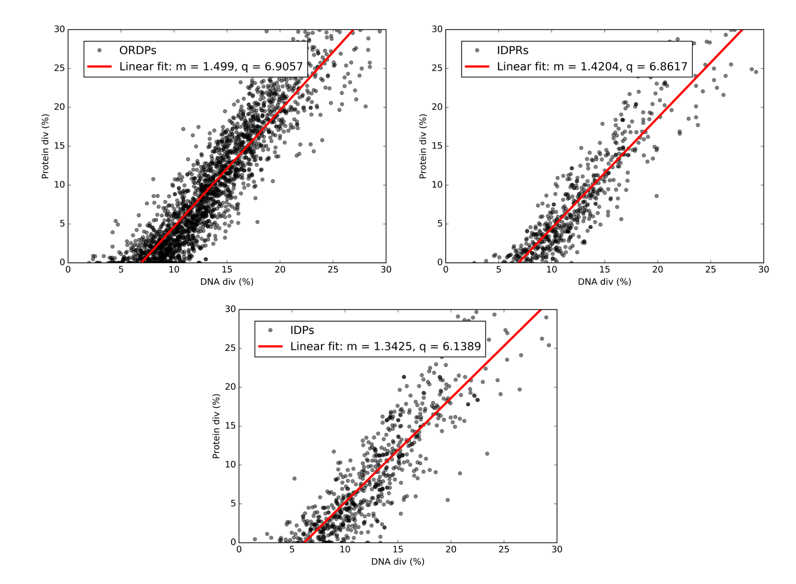


Macaca fascicularis Mus musculus


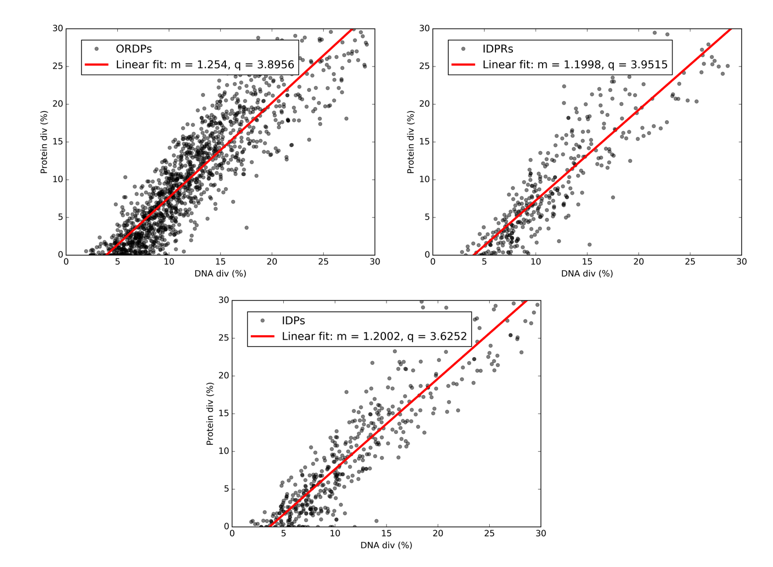

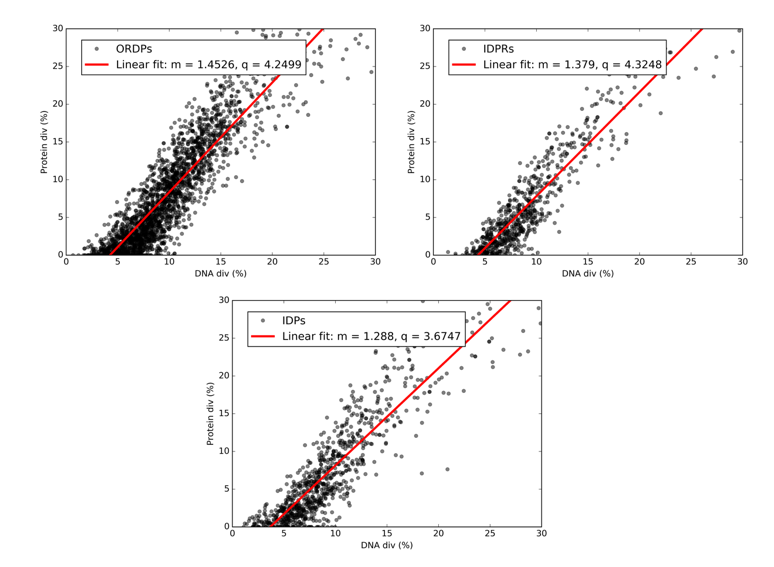


Ovis aries Panthera pardus


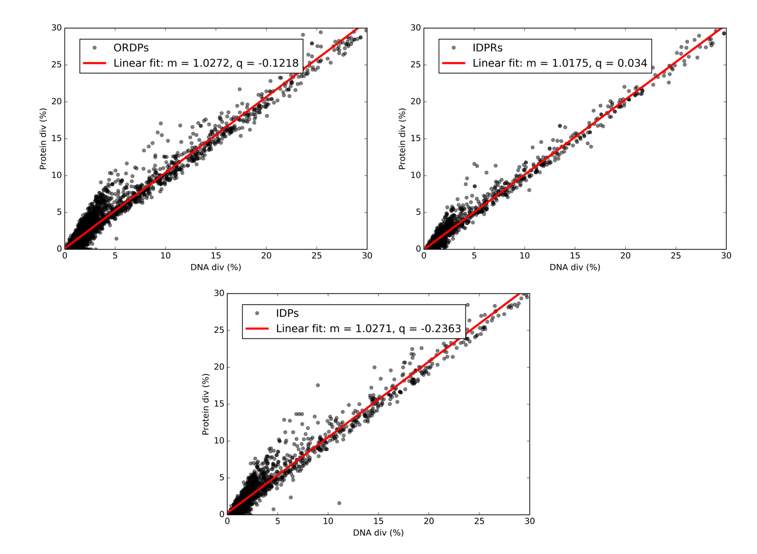

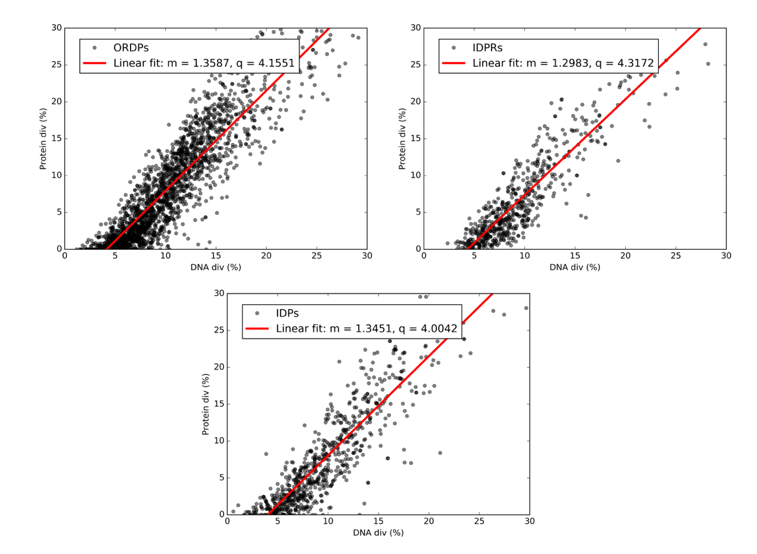


Pongo abelii Urocitellus parryii

**Figure S1: DNA divergence Vs. Protein divergence plots.** Relationship between nucleotide (DNA div) and amino acid (Protein div) sequence divergence obtained by confronting progressively human coding sequences (separated in ORDPs, IDPRs, and IDPs) with their homologs from 26 eukaryotes. Each point corresponds to an individual gene. In each panel, we report the best-fit line, together with the associated values of the slope (m) and the intercept (q) in the legend.


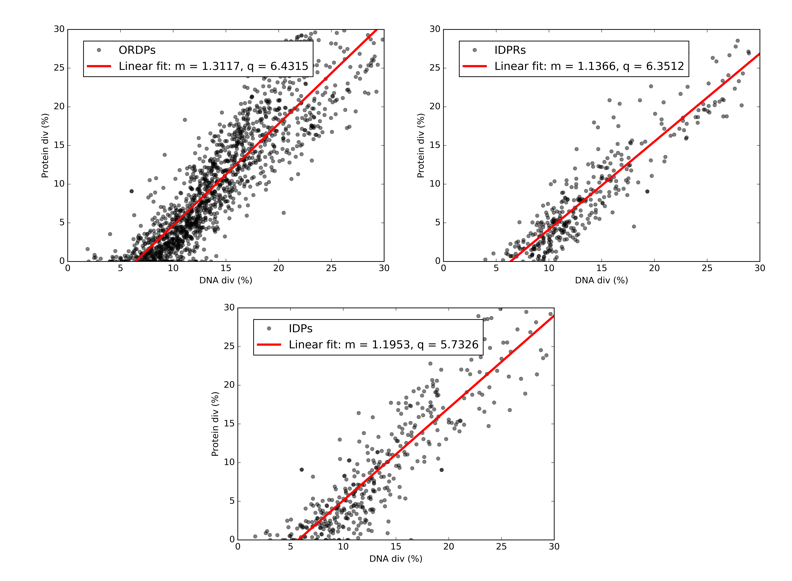

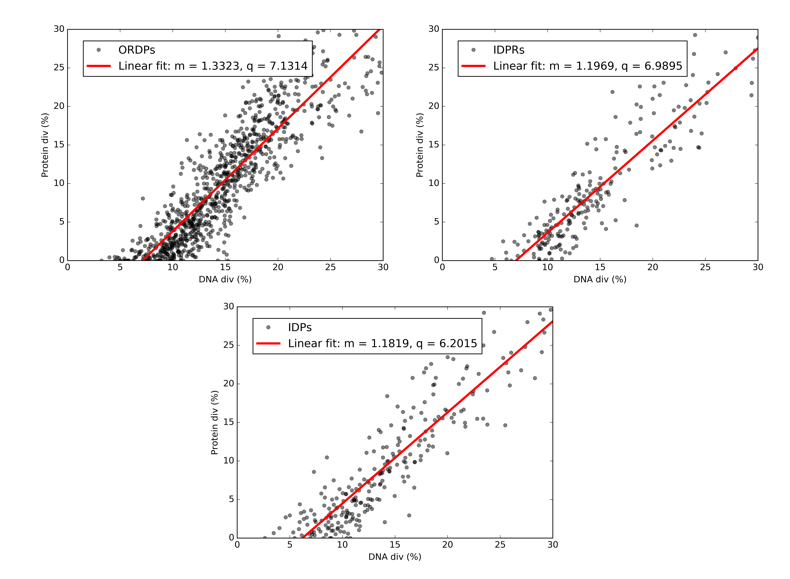


Mandrillus leucophaeus Ovis aries


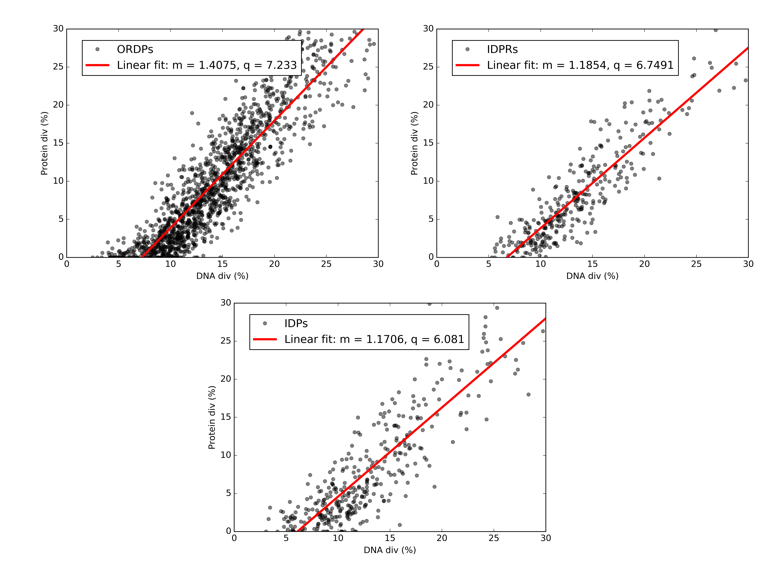

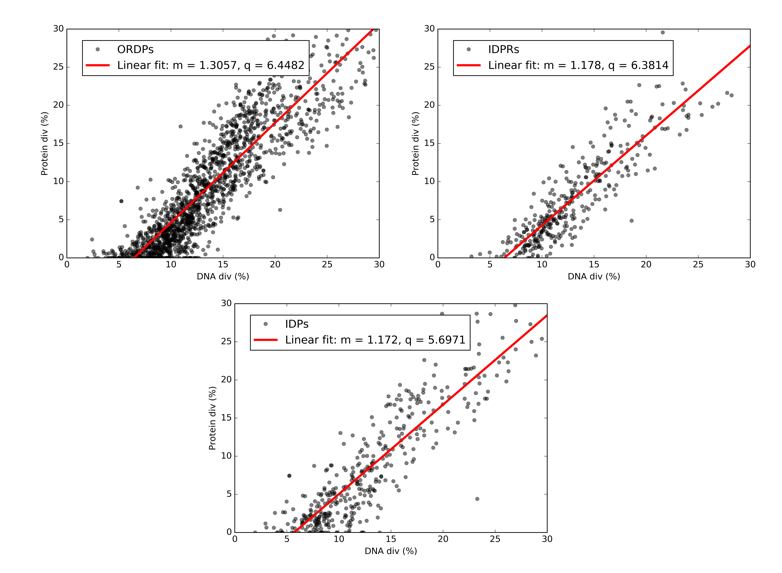


Vulpes Vulpes Pongo abelii


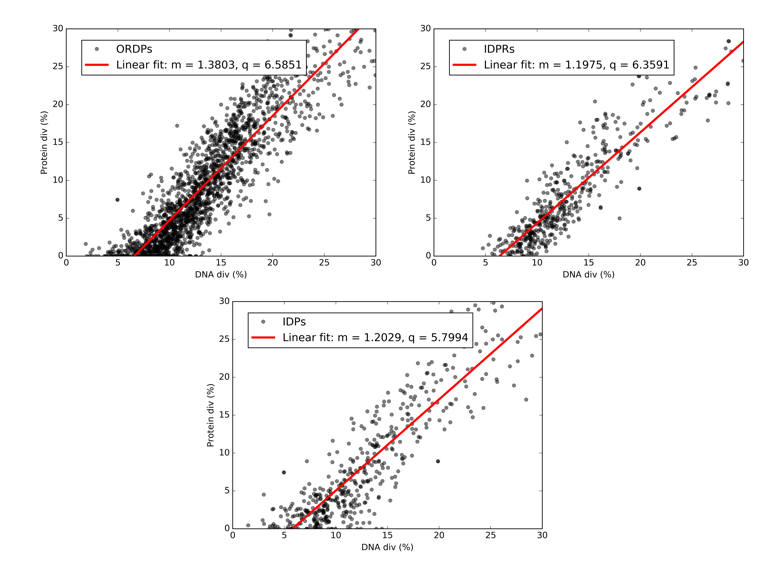

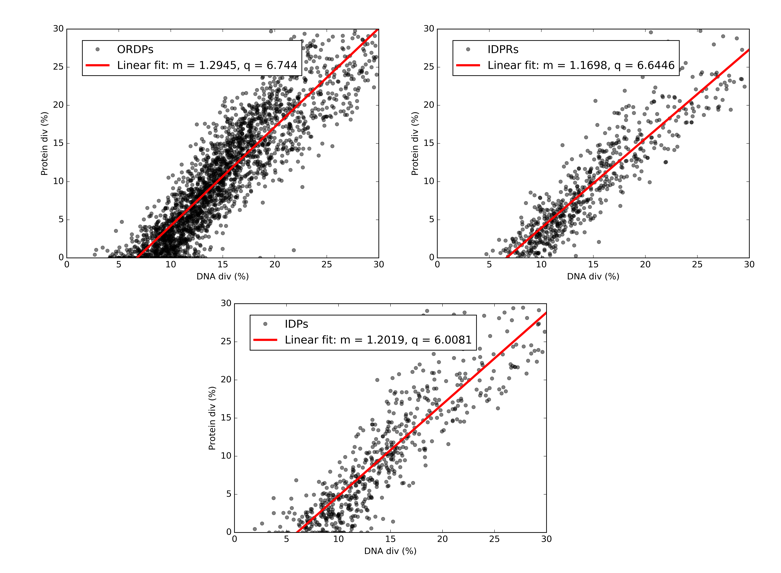


Pan paniscus Fukomys damarensis


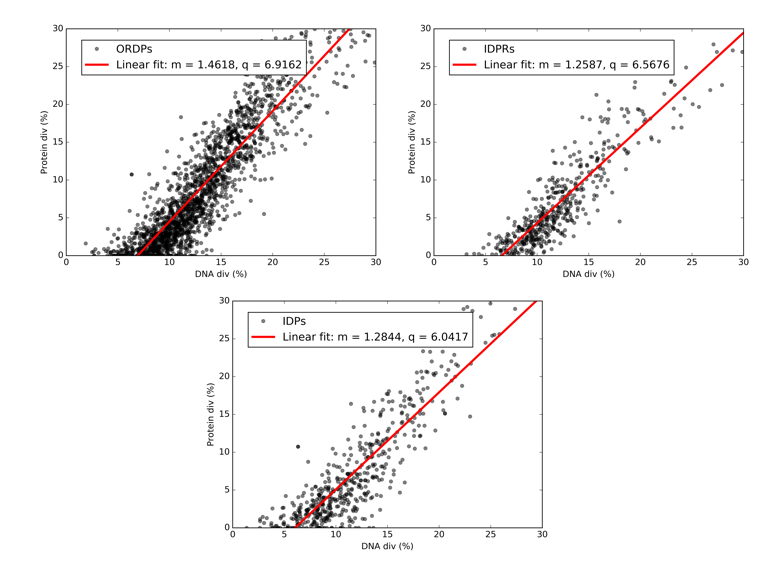

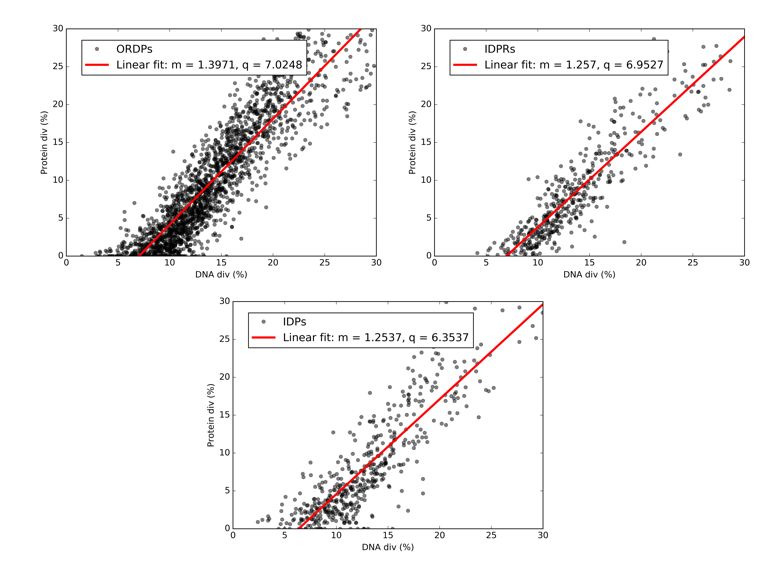


Macaca nemestrina Otolemur garnettii


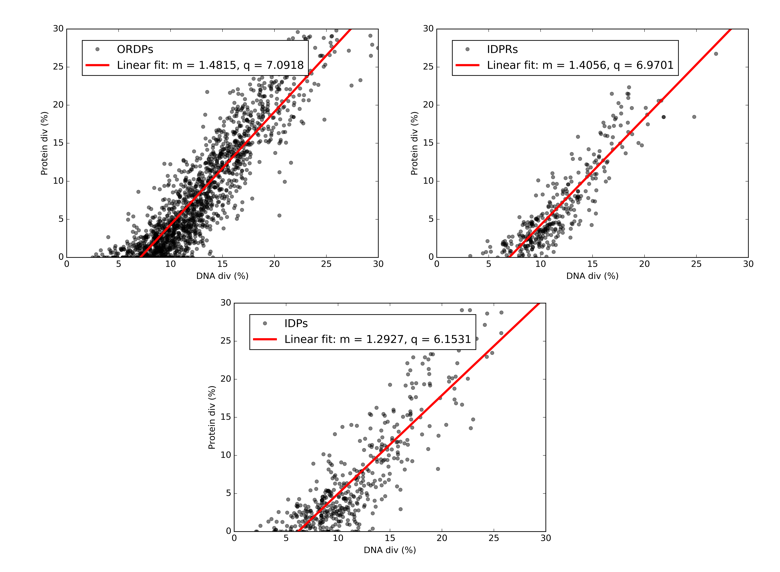

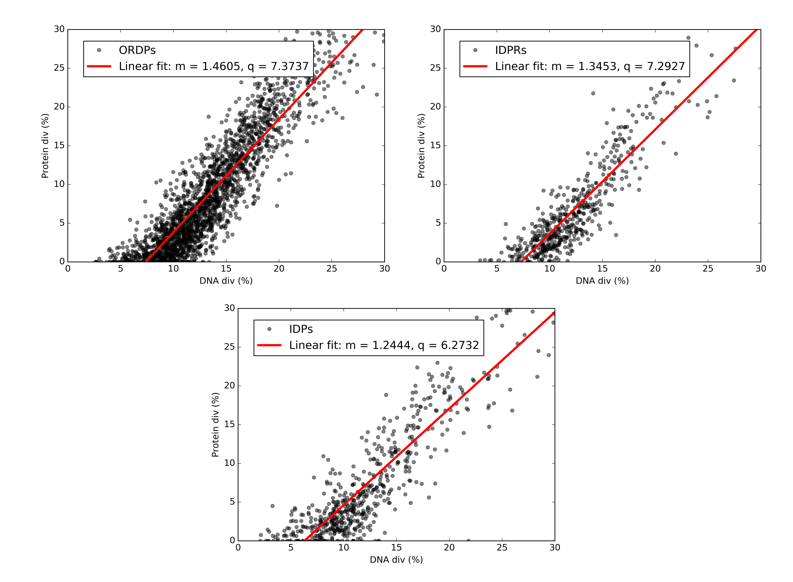


Equus caballus Panthera pardus


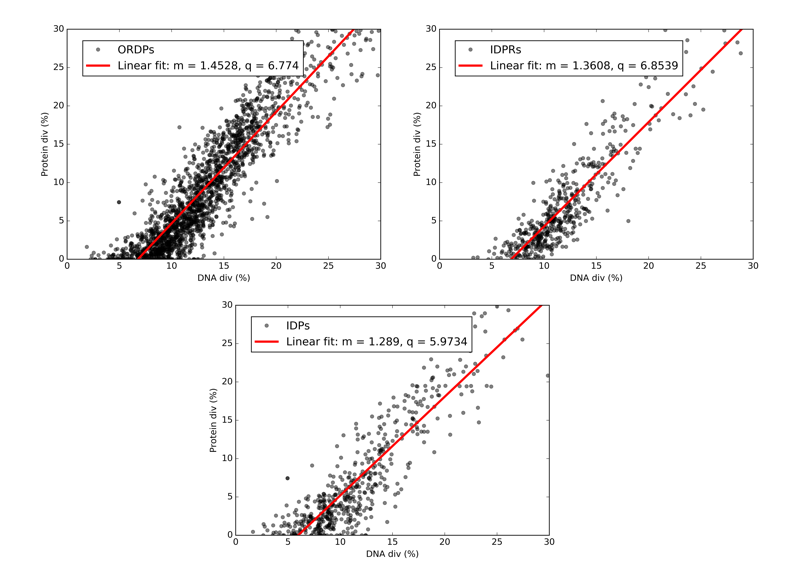

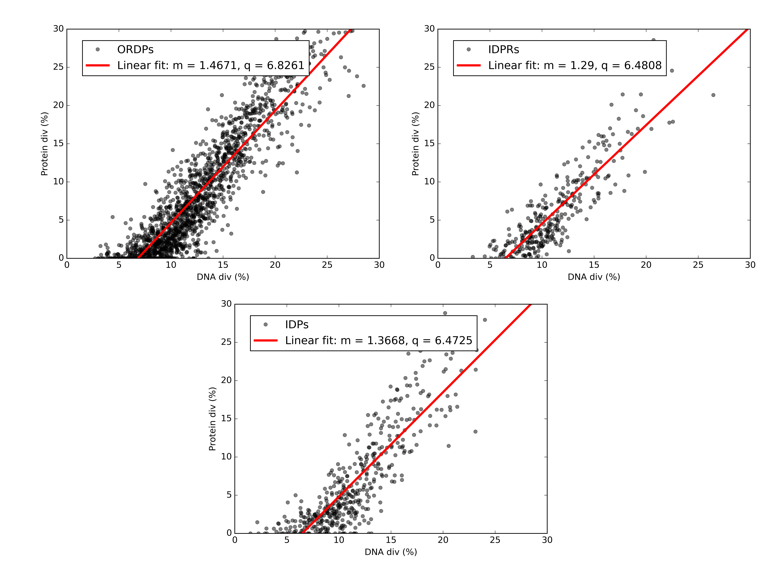


Pan troglodytes Urocitellus parryii


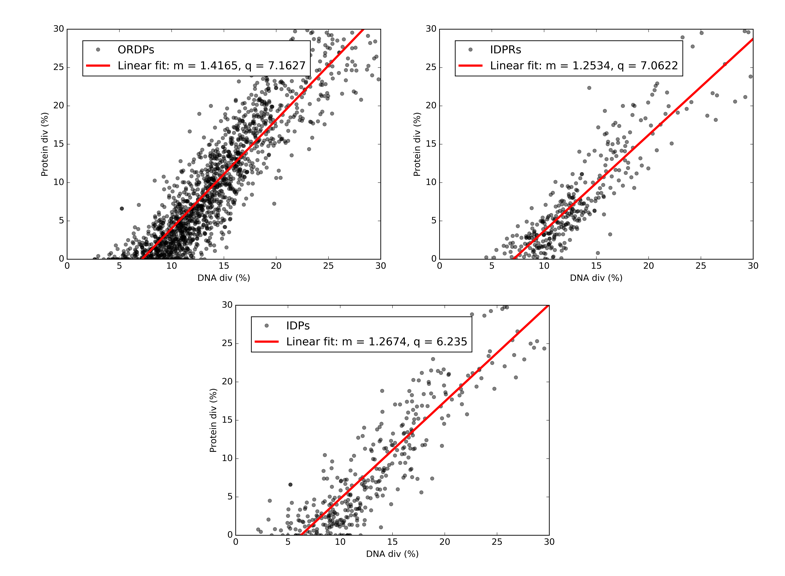

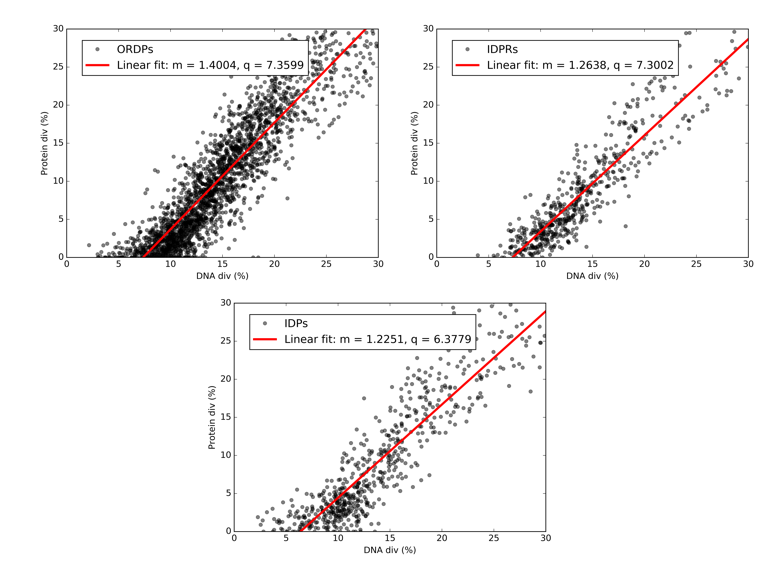


Panthera tigris altaica Octodon degus


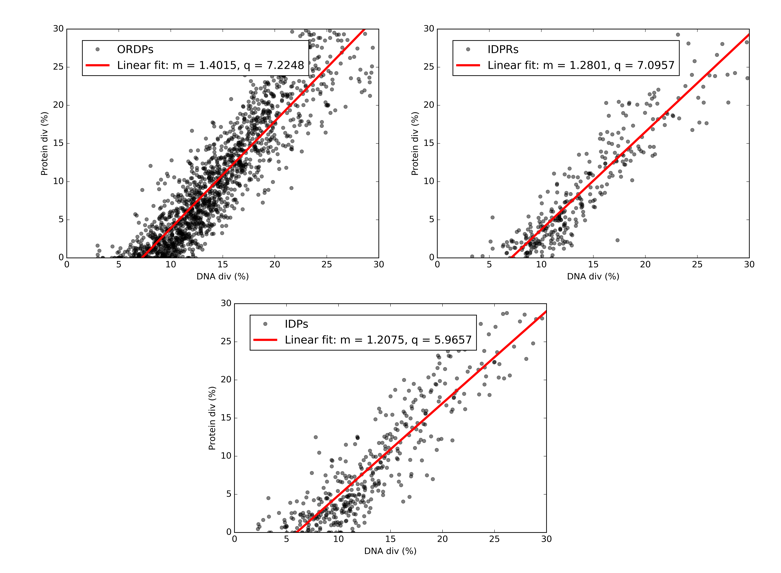

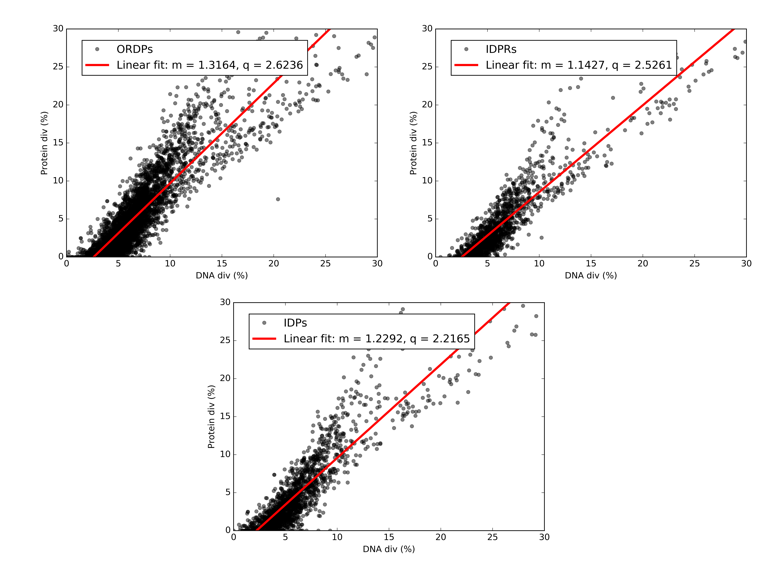


Ursus americanus Rattus norvegicus


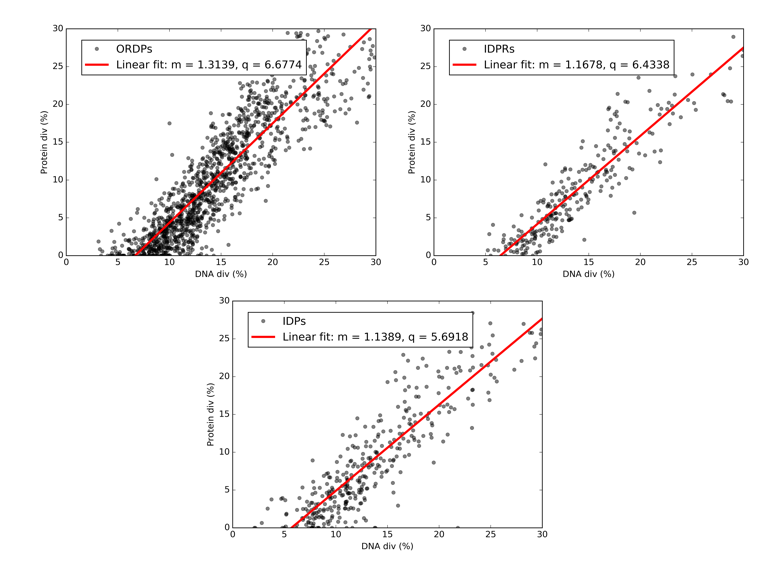

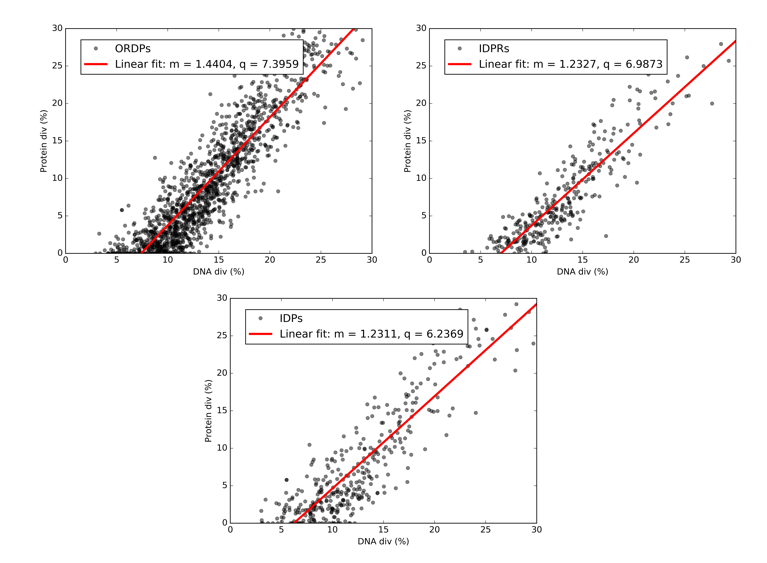


Canis familiaris Tursiops truncatus


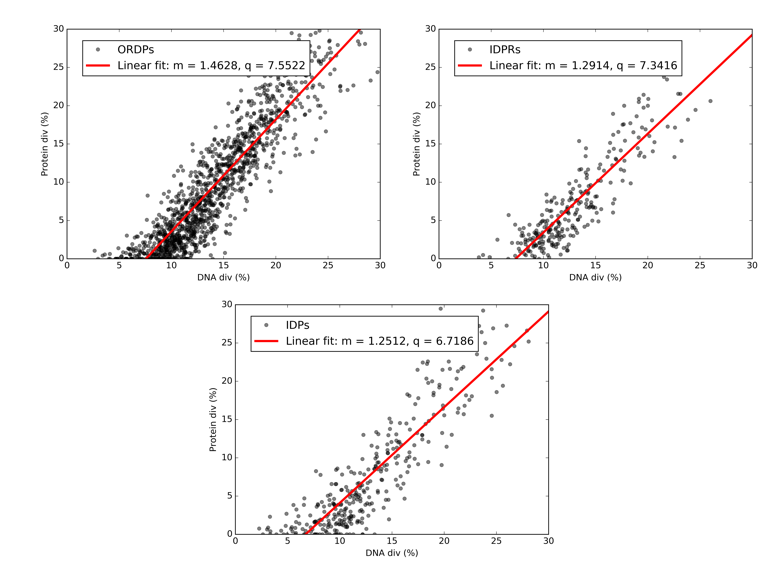

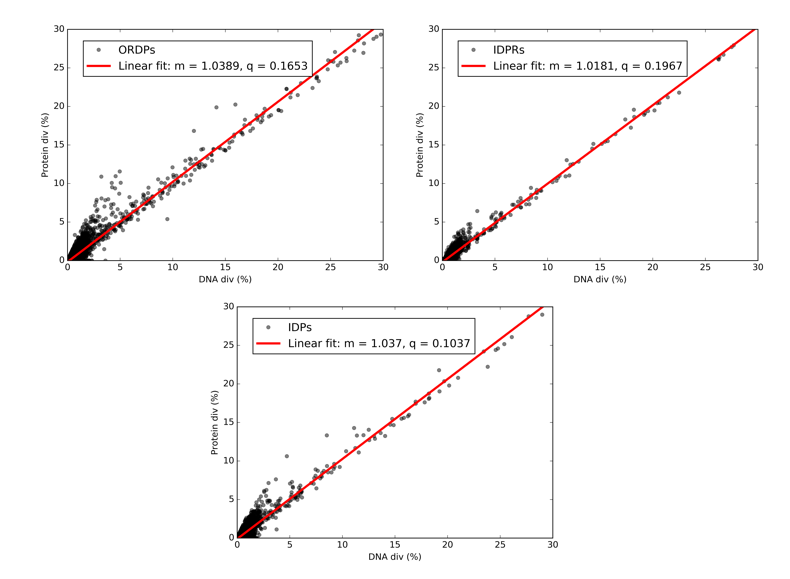


Bison bison bison Mus spretus


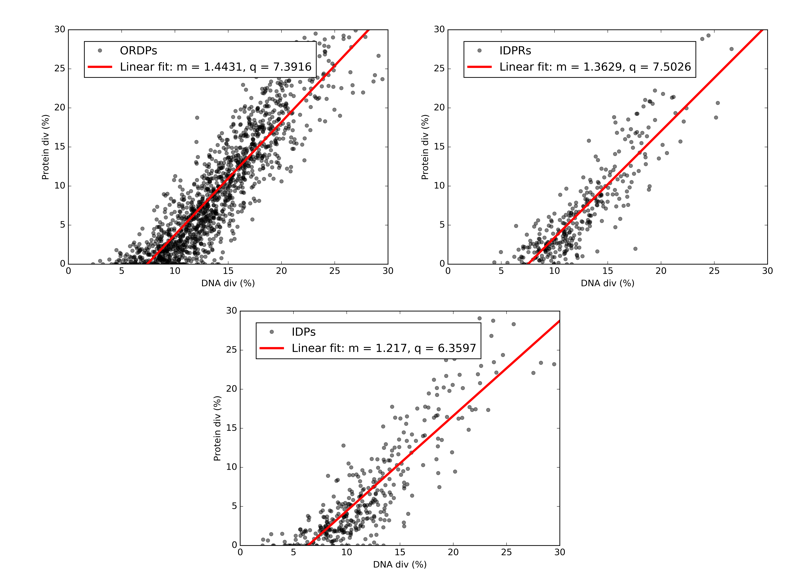

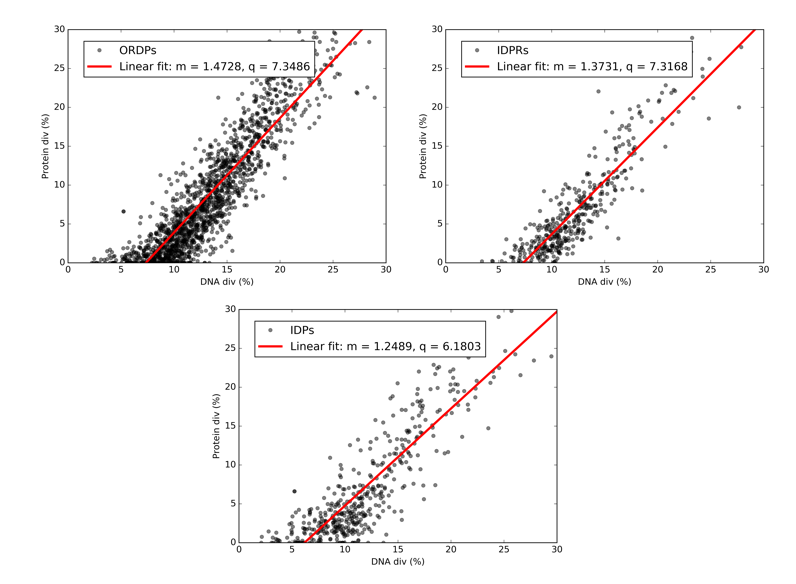


Sus scrofa Felis catus


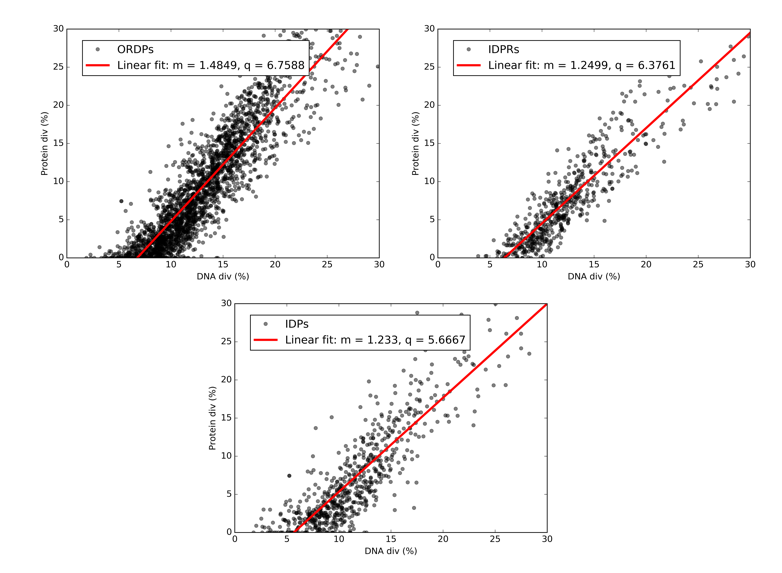

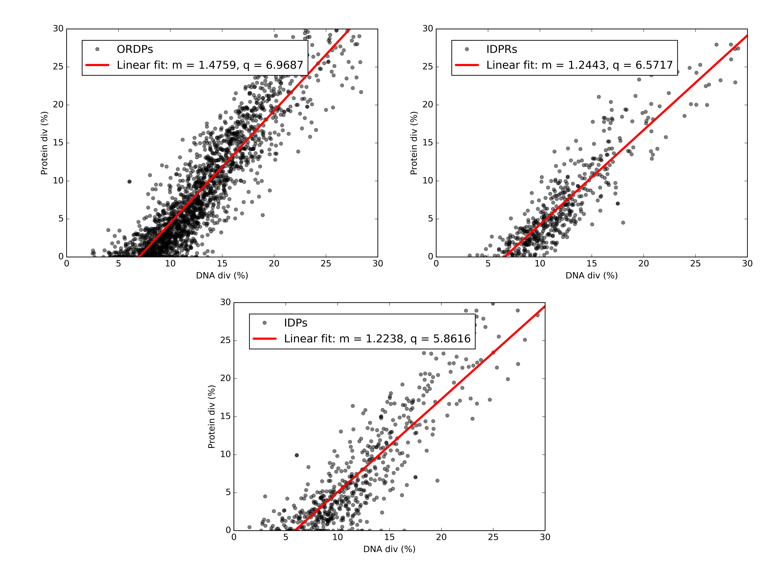


Castor canadensis Macaca fascicularis

**Figure S2: DNA divergence Vs. Protein divergence plots.** Relationship between nucleotide (DNA div) and amino acid (Protein div) sequence divergence obtained by confronting progressively coding sequences of Mus Musculus (separated in ORDPs, IDPRs, and IDPs) with their homologs from 26 eukaryotes. Each point corresponds to an individual gene. In each panel, we report the best-fit line, together with the associated values of the slope (m) and the intercept (q) in the legend.
